# Development of Indirect Competitive Immuno-Assay Method Using SPR Detection for Rapid and Highly Sensitive Measurement of Salivary Cortisol Levels

**DOI:** 10.3389/fbioe.2014.00015

**Published:** 2014-05-30

**Authors:** Yusuke Tahara, Zhe Huang, Tetsuro Kiritoshi, Takeshi Onodera, Kiyoshi Toko

**Affiliations:** ^1^Graduate School of Information Science and Electrical Engineering, Kyushu University, Fukuoka, Japan; ^2^Research and Development Center for Taste and Odor Sensing, Kyushu University, Fukuoka, Japan

**Keywords:** salivary cortisol, stress, surface plasmon resonance, self-assembled monolayer, immunosensor

## Abstract

The monitoring of salivary cortisol as a key biomarker of an individual’s stress response has been increasingly focused on. This paper describes the development of a novel cortisol immuno-assay method based on an indirect competitive method using a commercially available surface plasmon resonance instrument. The surface of an Au chip was modified with PEG6-COOH aromatic dialkanethiol self-assembled monolayers and hydrocortisone 3-(*O*-carboxymethyl) oxime (hydrocortisone 3-CMO) as a cortisol analog. A detection limit of 38 ppt range with a measurement range of 10 ppt–100 ppb was accomplished without the incubation of a mixing solution consisting of standard cortisol and an anti-cortisol antibody, and the time for quantification of cortisol concentration was 8 min from the sample injection. We experimentally compared our immuno-assay with a commercialized salivary cortisol enzyme-linked immunosorbent assay (ELISA) kit using human saliva samples. It was found that the results obtained by the cortisol immuno-assay had a good correlation with those obtained by ELISA assay (*R* = 0.96). Our findings indicate the potential utility of the cortisol immuno-assay for measurements of human salivary cortisol levels.

## Introduction

In the hypothalamus–pituitary–adrenal (HPA) axis, plasma, urinary, and salivary cortisol, key biomarkers of an individual’s psychological stress response, are being increasingly focused on in psychobiological stress research (Kirschbaum and Hellhammer, 2007; Olff et al., 2007). Many research groups have reported the relationship between the cortisol concentration and stress-related disorders, including irritable bowel syndrome (IBS) (Patacchioli et al., 2001), chronic fatigue syndrome (CFS) (Jerjes et al., 2006), and posttraumatic stress disorder (PTSD) (Inslicht et al., 2006; Johnson et al., 2008).

Salivary cortisol, in particular, is a major neuroendocrine correlator of the neuroendocrine stress response and has been increasingly studied as a stress biomarker (Hellhammer et al., 2009). Salivary cortisol levels ranged between 0.1 and 10 ppb (ng/ml) (Aardal and Holm, 1995). Our research group has focused on salivary cortisol and its analytical method (Tahara et al., 2010), because saliva can be collected sufficient volumes for biochemical analyses, with some exceptions such as dry mouth patients, regardless of the time and place by a non-invasive, and stress-free process without medical personnel. The whole salivary secretion rate has been reported approximately 1 ml/min (resting saliva) (Molander and Birkhed, 1981). The salivary cortisol level differs greatly among individuals and with circadian variation (Adam et al., 2006). Other research groups have reported that salivary cortisol levels are significantly lower in IBS patients (Patacchioli et al., 2001) and that there is a significant difference between depressed and healthy people with regard to salivary cortisol levels when waking and 30 min after waking (Alderling et al., 2008). However, immuno-assay-based clinical auto analytical instruments are expensive and their use is time-consuming. Conventional enzyme-linked immunosorbent assay (ELISA) in labs usually takes approximately 4 h to complete the analysis and multiple steps are required. Thus, the development of rapid, portable, and easy-to-use analytical devices designed to measure and rapidly report cortisol levels would have a great impact on future research and the treatment of people with stress-related disorders. Recently, various types of cortisol biosensors for point-of-care testing (POCT), such as a polyaniline based electrochemical immunosensor (Arya et al., 2011; Kaushik et al., 2013; Vasudev et al., 2013), an immunosensor with a fluid control mechanism (Yamaguchi et al., 2013), a single-walled carbon nanotubes (SWNT) chemiresistor transducer-based cortisol immunosensor (Tlili et al., 2011) have been proposed.

Our research group has developed a compact sensing system using surface plasmon resonance (SPR) immunosensors for the on-site detection of trinitrotoluene (MW: 227.1) (Mizuta et al., 2008; Onodera et al., 2009; Onodera, 2013). SPR immunosensors are realized by combining SPR sensors, which can be used to detect changes in the refractive index of the sensor surface with high-sensitivity, with an antigen–antibody interaction, resulting in high selectivity. However, cortisol, a steroid hormone, is a monovalent antigen with a low molecular weight (362.47). Therefore, the sandwich immuno-assay method cannot be applied. Other research groups have reported an SPR immunosensor using a direct competitive assay with primary and secondary antibodies used for the amplification of signals [response time: 15 min from sample injection, assay dynamic range: 91–900 ppt, detection limit (the blank less two SDs of the blank) using standard solution: 13 ppt] (Mitchell et al., 2009), using a direct assay with polycarboxylate-hydrogel-based coating for the antibody immobilization (response time: flowed over the sensors surface for 15 min, linear detection range: 5–154 ppb, detection limit using standard solution: 10 ppb) (Frasconi et al., 2009), as well as an SPR immunosensor using a cortisol-conjugated BSA-modified Au chip as a cortisol analog (response time: 15 min from sample injection, detection range: 1.5–10 ppb, detection limit using standard solution: 360 ppt) (Stevens et al., 2008).

In this study, we report a salivary cortisol immuno-assay method using a commercially available SPR instrument and a newly designed indirect competitive method for the quantitative evaluation of human psychosomatic stress. To this end, we modified a sensor surface with PEG6-COOH aromatic dialkanethiol self-assembled monolayers (SAMs) and hydrocortisone 3-CMO as a cortisol analog for highly sensitive, accurate, and rapid detection. Moreover, we compared the characteristics of the cortisol immuno-assay method a conventional ELISA assay using human saliva.

## Materials and Methods

### Reagents and chemicals

Mouse anti-cortisol monoclonal antibody (association constant *K*_A_ = 1.7 × 10^9^ M^−1^, cortisol antibody) was purchased from Abcam PLC (UK). As the cortisol analog, hydrocortisone 3-(*O*-carboxymethyl) oxime (hydrocortisone 3-CMO) was purchased from Sigma Aldrich (USA). PEG6-COOH aromatic dialkanethiol (PEG6-COOH) was purchased from SensoPath Technologies, Inc. (USA), and was used as a reagent to form SAMs on the chip surface. The Au sensor chip included in the SIA Kit, the *N*-ethyl-*N*-(3-dimethylaminopropyl) carbodiimide hydrochloride (EDC) included in an amine coupling kit, the *N*-hydroxysuccinimide (NHS) for sensor surface fabrication, a borate buffer (pH 8.5, 10 mM disodium tetraborate, 1 M NaCl), and 50 mM NaOH were purchased from GE Healthcare Bio-Science AB (Sweden). Ethylenediamine was purchased from Wako Pure Chemical Industries, Ltd. (Japan). An ELISA kit was used as a conventional salivary cortisol detection method, and Salimetrics oral swabs, used as a salivary collection material were purchased from Salimetrics LLC (USA). Water purified using a Milli-Q integral water purification system was used as the solvent. All aqueous solutions were prepared with Milli-Q water obtained from a Milli-Q system (Millipore Corporation, USA).

### Modification of sensor chip surface

**Figure 1:**
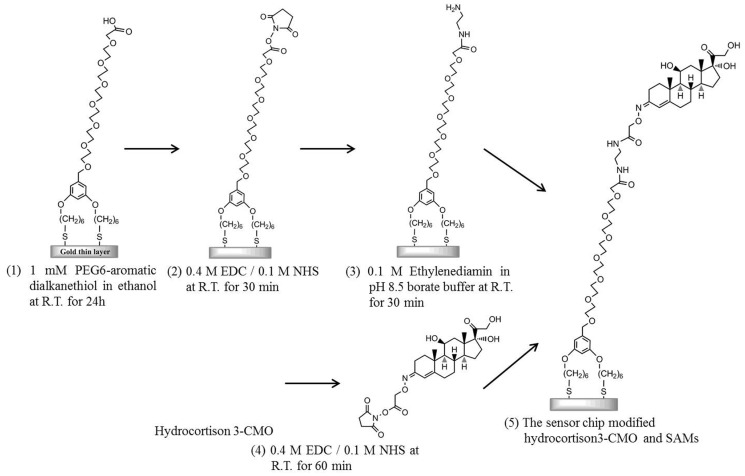
**Fabrication procedure of the sensor chip modified with cortisol analog**.

As a preliminary cleaning step, an Au sensor chip covered with a 50-nm-thick layer of unmodified gold was first ultrasonically cleaned in acetone for 10 min, ethanol for 2 min, and 2-propanol for 2 min. Subsequently, the sensor surface was cleaned in standard clean solution (a mixture of ammonia solution, hydrogen peroxide solution, and pure water with a ratio of 1:1:5) heated to 90°C for 20 min. Modification of the sensor chip surface was performed as follows (Figure 1): the sensing surface of the Au sensor chip was immersed in 1 mM PEG6-COOH (Lahiri et al., 1999) in ethanol for 24 h to form SAMs on the sensor surface. After cleaning with ethanol by ultrasonication, a mixture solution consisting of 0.2 M EDC in water and 50 mM NHS in water was added dropwise onto the sensor surface and incubated for 30 min to activate the terminal carboxyl group. Next, ethylenediamine in the pH 8.5 borate buffer was added dropwise onto the sensor surface and incubated for 30 min to induce amine coupling by covering the terminal carboxyl group of the SAM into an amino group. Simultaneously, 10 mM hydrocortisone 3-CMO in DMF with a carboxyl group as a cortisol analog, 0.4 M EDC in water, and 0.1 M NHS in DMF were mixed with a volume ratio of 1:1:1 and incubated for 60 min to activate the carboxyl group of the cortisol analog. The mixed solution was added dropwise onto the sensor surface and incubated for 60 min. Finally, the cortisol analog was bound to ethylenediamine immobilized on the film surface as a result of amine coupling.

### Cortisol detection using SPR immunosensor by indirect competitive assay

Cortisol detection was performed by SPR using a Biacore J system (GE Healthcare, Japan). An indirect competitive assay was applied for cortisol measurements. 10 mM 4-(2-hydroxyethyl)-1-piperazineethanesulfonic acid (HEPES) buffered saline (HBS, 150 mM NaCl, 0.05% Tween 20, pH 7.4) was used as the running buffer.

When the antibody solution is injected onto the chip surface, the antibodies bind to the cortisol analog immobilized onto the chip surface, resulting in an increased sensor response. The time from injection to reach the sensor surface is within a few seconds. The increase in the sensor response (*Δθ*_0_) represents the number of antibodies bound to the chip surface. When a mixture of antibodies and cortisol is injected onto the chip surface, the antibodies that have already bound to the cortisol do not bind to the cortisol analog on the chip surface. Namely, the cortisol in the solution inhibits the antibodies from binding to the chip surface. Therefore, the concentration of antibodies bound to the chip surface decreases from that when only the antibodies are injected (*Δθ*_1_). It is considered that *Δθ*_1_ decreases as the percentage of cortisol in the mixture increases. Here, the relative change in the concentration of antibodies bound to the chip surface, i.e., the bound percentage, is calculated by

(1) $$\left(\Delta \theta_{1}∕\Delta \theta_{0}\right)\times 100.$$

The smaller the bound percentage, the larger the concentration of antibodies bound to cortisol. Antibodies and cortisol, each with a concentration of twice the final concentration, were prepared for the measurement.

A mixture of the antibody solution and HBS buffer was injected onto the chip surface for 5 min, followed by the injection of a regeneration solution for 3 min at a flow rate of 15 μl/min to regenerate the chip surface. Subsequently, the antibody and cortisol solutions were mixed with a ratio of 1:1 and injected onto the chip surface for 5 min at a flow rate of 15 μl/min. Next, to regenerate the sensor surface, 50 mM NaOH was allowed to flow over the sensor surface for 3 min at a flow rate of 15 μl/min.

### Calculation of association constant

Antibody solutions with different concentrations (0.1–2.0 ppm) were injected onto the three sensor chips with the cortisol analog immobilized on the surface for 5 min at 30 μL/min. After the injection, the sensor chip was incubated for 5 min to allow spontaneous dissociation. The obtained sensorgram was used to calculate the reaction rate constants. BIAevaluation version 4.1 software (GE Healthcare Bio-Science) was used for analysis as well as the “bivalent with mass transfer” fitting model, which assumes that antibodies undergo bivalent binding and that the association of antibodies occurs under mass transport limitation. Five sensorgrams with six concentrations of the antibody solution were simultaneously analyzed by the fitting program to calculate the association rate constant *K*_a_ and the dissociation rate constant *K*_d_. Using these constants, the association constant *K*_A_ (=*K*_a_/*K*_d_) was calculated.

### Calibration curve for cortisol detection

To construct a calibration curve, the sensor response was measured three times for each cortisol solution with concentrations of 0.01–100 ppb (1 ppb = 1 μg/l = 2.8 nM) following the above procedure. A bound percentage equal to a value three times the standard deviation was adopted as the detection limit. BIAevaluation version 4.1 software (GE Healthcare Bio-Science) was used for calculating the calibration curves.

### Collection and preparation of human saliva samples

To collect human saliva, four healthy young Japanese adults (25 ± 1.2 years) were enrolled in this study. The study protocol was approved by the Ethical Committee of Kyushu University. The subjects were not allowed to take any food or drinks, except for water, for 1 h prior to the collection. To clean the oral cavity, each subject rinsed their mouth. To collect whole saliva, the subjects inserted a salimetrics oral swab into the oral cavity under the tongue for about 3 min. Saliva was collected at 10:00 a.m., 2:00, and 6:00 p.m. The saliva was separated from the oral swab by centrifugalization at 3,000 rpm for 15 min. Then, the saliva samples were stored at −20°C until analysis.

## Results and Discussion

### Evaluation of association constants of the cortisol analog to the antibody

In order to confirm that the hydrocortisone 3-(*O*-carboxymethyl) oxime (hydrocortisone 3-CMO) can be applied to this indirect competitive assay as a cortisol analog, the strength of association constants (*K*_A_) of the modified sensor chip was analyzed. From the calculation result, *K*_A_ was 3.07 × 10^7^ M^−1^ and lower than *K*_A_ between cortisol and the cortisol antibody (*K*_A_ = 1.7 × 10^9^ M^−1^). It indicated that the cortisol analog-modified sensor chip can be applied to the indirect competitive assay.

### Calibration curve

**Figure 2:**
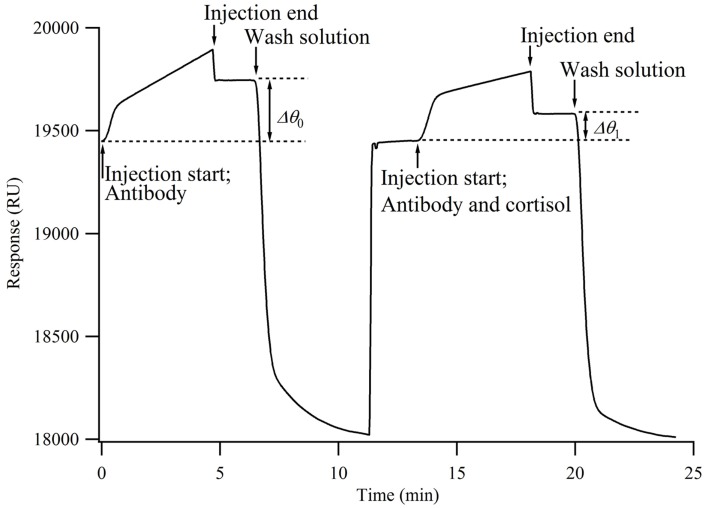
**Surface plasmon resonance sensorgram obtained from indirect competitive assay**.

A calibration curve for the cortisol measurement was obtained using a standard cortisol solution consisting of cortisol with various concentrations, HBS, and 2.5% ethanol (*n* = 3). Figure 2 shows an SPR sensorgram obtained by this measurement. When a mixture of 600 ppb antibody and HBS (final concentration, 300 ppb) was injected onto the chip, the change in the sensor response (*Δθ*_1_) was 207.4 ± 4.7 RU (*n* = 18). Here, 1000 RU corresponds to approximately 1 ng/mm^2^, and the amount of immobilized antibody was 207.4 pg/mm^2^. Dissociation of the bound antibody from the sensing surface is required for the next immunocycle. These results indicate that the regeneration conditions are suitable, because the sensor responses (*Δθ*_1_) were nearly unchanged.

**Figure 3:**
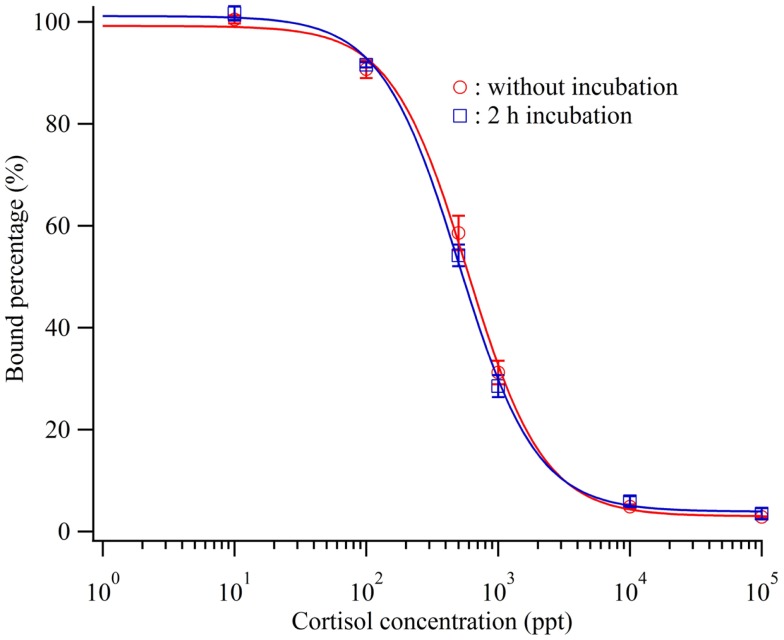
**Calibration curve of the cortisol biosensor with or without incubation before injection of cortisol standard cortisol and cortisol antibody**. 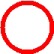 ; without incubation, 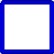 ; 2 h incubation.

Before injection onto the sensor surface, each mixture of the antibody and cortisol solutions was incubated for 2 h. A mixture solution containing 600 ppb antibody and the each standard cortisol solution was applied for 5 min after incubation for 2 h or without incubation before injection. Figure 3 shows the obtained calibration curves. Even without incubation, a calibration curve similar to that for the case of 2 h incubation was obtained. The detection limits of the calibration curves in the case of 2 h incubation and no incubation were 72.8 and 38.0 ppt, respectively. Alderling et al. reported that the cortisol level in human saliva ranges from approximately 2–20 nM (0.7–7.1 ppb) with circadian variation. Thus, this cortisol immuno-assay method does not need sample incubation, antibody, and cortisol solution, before sample injection to monitor human cortisol levels. Here, *Δθ*_1_ for cortisol quantification was determined approximately 8 min after sample injection and rapid compared with other reports. Moreover, it can be used to analyze the cortisol concentrations in blood plasma and urine, which are approximately 10 times higher than that in saliva, upon controlling the sensitivity.

### Comparison of SPR and ELISA assay

**Figure 4:**
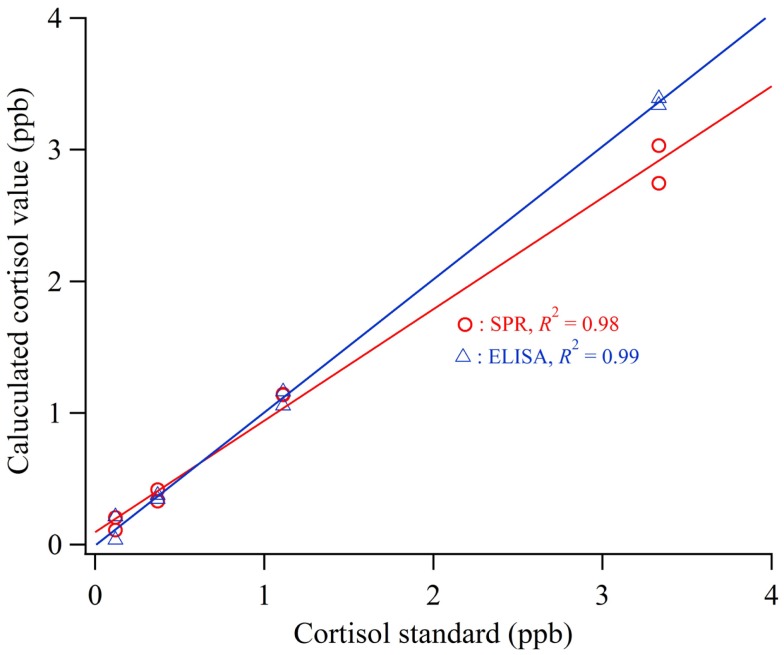
**Calculated cortisol values using the sensor and ELISA applied to cortisol standard solution**.

To evaluate the accuracy of the cortisol biosensor for detecting cortisol in human saliva, we carried out a comparison with a conventional cortisol ELISA kit. The calibration curve for the cortisol measurement was applied using standard cortisol solution included in a commercialized ELISA kit (*n* = 2). Figure 4 shows the calculated cortisol values using the sensor and ELISA applied to cortisol standard solution (0.12–3 ppb). The values of determination (*R*^2^) of the sensor and ELISA were 0.98 and 0.99, respectively.

In the ELISA assay, after the human saliva samples were thawed at room temperature, they were centrifuged at 3,000 rpm for 15 min and the supernatant sample was used in the ELISA assay. The measurement was performed following the manufacturer’s manual. The cortisol concentration ranged between 1.3 and 4.1 ppb (3.7 and 11.2 nM). These results were shown to be in reasonable agreement with those previously reported (Kirschbaum and Hellhammer, 2007; Hellhammer et al., 2009). In the SPR assay, after the human saliva samples were thawed at room temperature, they were centrifuged at 13,000 rpm using a Microcon centrifugal filter unit membrane (molecular weight cutoff 30 kDa, Millipore Corporation, USA) and mixed with HBS with a volume ratio of 1:3 to remove proteins. The SPR assay was carried out following the above procedure. Here, the cross reactivity of the anti-cortisol antibody to steroid hormones in saliva such as progesterone, testosterone, estradiol, and estriol was lower than 0.1% according to the data sheet of the manufacturers.

**Figure 5:**
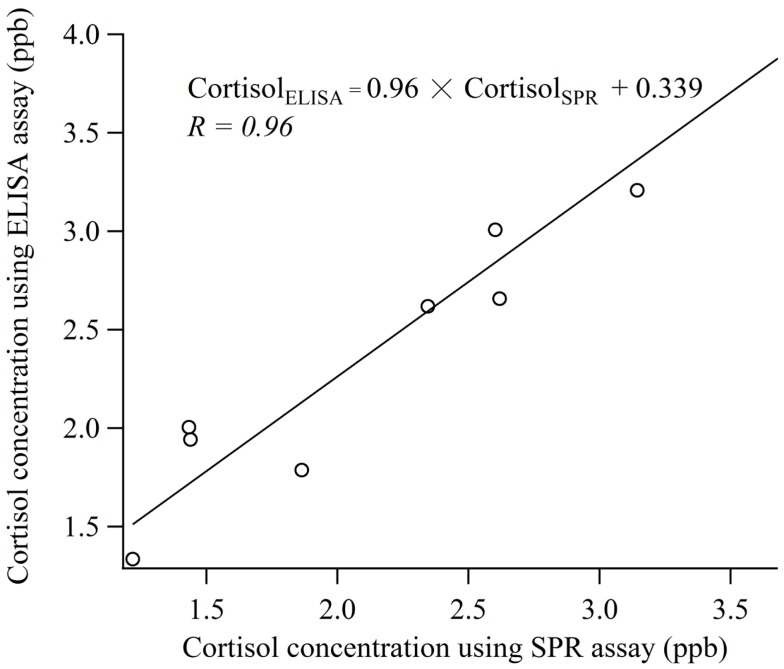
**Comparison of the measurement values obtained with cortisol biosensor and ELISA using human saliva samples**.

Figure 5 shows the results of the comparative experiment using human saliva samples. Linear regression analysis was used to determine the relationship between the measurement values obtained by the biosensor and the ELISA. The correlation coefficient was 0.96 and the regression equation relative the values of cortisol obtained using the SPR (Cortisol_SPR_) and ELISA (Cortisol_ELISA_) was Cortisol_ELISA_ = 0.96 × Cortisol_SPR_ + 0.339. The values of slope ±95% confidence interval (CI) and intercept ±95% were 0.96 ± 0.239 and 0.339 ± 0.572, respectively. Thus, it is indicated that the cortisol immuno-assay has good correlation and is capable of performing with similar accuracy to the conventional ELISA. It is necessary to develop the technology for improving sample preparation method in a short time.

## Conclusion

A cortisol immuno-assay employing a newly designed indirect competitive method and a commercially available SPR instrument with high-sensitivity, rapid, simple, and label-free measurements was invented for salivary cortisol measurements to replace time-consuming laboratory analysis and facilitate the POCT of salivary cortisol. The Au surface of a sensor chip was modified with PEG6-COOH aromatic dialkanethiol using hydrocortisone 3-CMO as a cortisol analog. A detection limit of 38 ppt was achieved by the sensor and the response time was 5 min from the sample injection. Moreover, because there was no effect of the incubation time of standard cortisol and cortisol antibody, cortisol measurement was possible without incubation. We experimentally compared our immuno-assay method using the commercially available SPR instrument with a commercialized salivary cortisol ELISA kit using human saliva samples. The results obtained by the cortisol biosensor had a good correlation with those obtained by ELISA assay (*R* = 0.96). Therefore, the measurement results suggest that the biosensor can be used to monitor salivary cortisol levels of human saliva samples. Moreover, it can be used to analyze the cortisol concentrations in blood plasma and urine.

## Conflict of Interest Statement

The authors declare that the research was conducted in the absence of any commercial or financial relationships that could be construed as a potential conflict of interest.
